# *Salmonella* Typhimurium Lacking YjeK as a Candidate Live Attenuated Vaccine Against Invasive *Salmonella* Infection

**DOI:** 10.3389/fimmu.2020.01277

**Published:** 2020-06-23

**Authors:** Soyeon Park, Bogyo Jung, Eunsuk Kim, Seong-Tshool Hong, Hyunjin Yoon, Tae-Wook Hahn

**Affiliations:** ^1^Department of Veterinary Medicine and Institute of Veterinary Science, Kangwon National University, Chuncheon, South Korea; ^2^Department of Molecular Science and Technology, Ajou University, Suwon, South Korea; ^3^Department of Biomedical Sciences and Institute for Medical Science, Chonbuk National University Medical School, Jeonju, South Korea

**Keywords:** *Salmonella* Typhimurium, live attenuated vaccine, YjeK, elongation factor P, non-typhoidal *Salmonella*, immune protection, virulence

## Abstract

Non-typhoidal *Salmonella* (NTS) causes gastrointestinal infection, which is commonly self-limiting in healthy humans but may lead to invasive infection at extraintestinal sites, leading to bacteremia and focal systemic infections in the immunocompromised. However, a prophylactic vaccine against invasive NTS has not yet been developed. In this work, we explored the potential of a Δ*yjeK* mutant strain as a live attenuated vaccine against invasive NTS infection. YjeK in combination with YjeA is required for the post-translational modification of elongation factor P (EF-P), which is critical for bacterial protein synthesis. Therefore, malfunction of YjeK and YjeA-mediated EF-P activation might extensively influence protein expression during *Salmonella* infection. *Salmonella* lacking YjeK showed substantial alterations in bacterial motility, antibiotics resistance, and virulence. Interestingly, deletion of the *yjeK* gene increased the expression levels of *Salmonella* pathogenicity island (SPI)-1 genes but decreased the transcription levels of SPI-2 genes, thereby influencing bacterial invasion and survival abilities in contact with host cells. In a mouse model, the Δ*yjeK* mutant strain alleviated the levels of splenomegaly and bacterial burdens in the spleen and liver in comparison with the wild-type strain. However, mice immunized with the Δ*yjeK* mutant displayed increased Th1- and Th2-mediated immune responses at 28 days post-infection, promoting cytokines and antibodies production. Notably, the Th2-associated antibody response was highly induced by administration of the Δ*yjeK* mutant strain. Consequently, vaccination with the Δ*yjeK* mutant strain protected 100% of the mice against challenge with lethal invasive *Salmonella* and significantly relieved bacterial burdens in the organs. Collectively, these results suggest that the Δ*yjeK* mutant strain can be exploited as a promising live attenuated NTS vaccine.

## Introduction

Non-typhoidal *Salmonella* (NTS) is one of the main causative agents of foodborne gastrointestinal diseases in humans (1). NTS infections in the gastrointestinal tract are generally mild, manifesting with symptoms such as abdominal cramps, nausea, diarrhea, mild fever, vomiting, dehydration, and/or headache (2, 3). However, NTS is often invasive and can enter the bloodstream with very serious consequences, including sepsis, pneumonia, meningitis or osteomyelitis, hepatosplenomegaly, and/or respiratory symptoms (3–5). Notably, invasive NTS (iNTS) is fatal to young children as well as to individuals suffering from malnutrition and immunocompromised patients, including those with HIV/AIDS and organ recipients (6). According to Branchu et al., ~3 million cases and 700,000 deaths per year in sub-Saharan Africa were associated with co-infection of iNTS (7). Despite the serious challenge of NTS infection, there is currently no NTS vaccine available for humans. Therefore, development of an NTS vaccine is considered one of the most upmost issues in healthcare at present.

Among the various types of vaccines developed to date, an attenuated vaccine, which is a live pathogen with its virulence attenuated, represents one of the most successful vaccines currently used. The best known examples of live attenuated vaccines include the oral polio vaccine, measles vaccine, and Bacillus Calmette–Guerin vaccine derived from *Mycobacterium bovis*. These vaccines have proven to exert good protective effects against infections of the targeted pathogens (8). These attenuated vaccines often show superior efficacy compared to other types of vaccines, including inactivated, subunit, recombinant, polysaccharide, and conjugated vaccines, suggesting that a live attenuated vaccine is a promising strategy for combatting certain infectious diseases (9). Therefore, it would be logical to develop a live attenuated vaccine against iNTS.

The success of a live attenuated vaccine depends on the nature of the bacterial strain with the virulence attenuated but immunogenicity maintained. Here, we showed that the deletion of *yjeK* gene attenuated the virulence of *Salmonella enterica* serovar Typhimurium without losing its immunogenicity. Animal experiments also showed that the attenuated Δ*yjeK S*. Typhimurium protected mice against subsequent iNTS infection efficiently, indicating that *S*. Typhimurium lacking YjeK could be developed as an ideal attenuated vaccine against NTS infection.

The *Salmonella yjeK* (or *epmB*) and *yjeA* (or *epmA, poxA, genX*) genes encode a truncated form of lysine-2,3-aminomutase and a lysyl-tRNA synthetase, respectively, which participate in the post-translational modification pathway of bacterial elongation factor P (EF-P). The proposed mechanism is as follows: YjeK first converts _L_-lysine to _D_-β-lysine, and YjeA then activates _D_-β-lysine to AMP- _D_-β-lysine to transfer the β-lysyl moiety to the ε-amino group of the Lys-34 residue of EF-P (10, 11). EF-P activated by this YjeK and YjeA-mediated β-lysylation stimulates *N*-formylmethionyl-puromycin synthesis in *Escherichia coli*, indicating an important role of EF-P activation in the first peptide bond formation during protein synthesis (12, 13). *E. coli* and *Salmonella* with impaired YjeK and YjeA-mediated EF-P activation suffer from growth defects and are susceptible to various physical and chemical stressors (14, 15). In *Salmonella*, the lack of YjeA was also shown to influence the production of *Salmonella* pathogenicity island 1 (SPI-1)-associated virulence factors (14, 16).

Given the extensive influence of EF-P activation in bacterial physiology, we explored the possibility of developing a live attenuated NTS vaccine by manipulating *Salmonella enterica* serovar Typhimurium virulence via the coordinated action between YjeK, YjeA, and EF-P without compromising its immunogenicity.

## Materials and Methods

### Bacterial Strains, Plasmids, and Growth Conditions

The bacterial strains and plasmids used in this study are listed in Table S1. The wild-type strain *Salmonella enterica* serovar Typhimurium 1120 (ST1120) isolated in Korea was used as a control strain in all experiments (17). In the animal challenge experiments, ST2173, a *S*. Typhimurium 14028 strain transformed with the plasmid pBBR1-MCS4 was used (18) containing an ampicillin resistance gene. The bacterial strains were cultivated in Luria-Bertani (LB) broth (Duchefa, Haarlem, Netherlands) at 37°C with ampicillin (Duchefa) at 150 μg/ml when required, unless otherwise specified. All antibiotics were purchased from Sigma-Aldrich (St. Louis, MO, USA). Bacterial biochemical characteristics were identified using the API 20E kit (bioMérieux, Inc., Durham, NC, USA). An ST1120 mutant strain lacking the *yjeK* gene was generated through site-directed mutagenesis using the λ red recombination method with plasmids pTP233, pKD3, and pCP20 (Table S1). The detailed procedure is described in a previous study (19, 20). In brief, ST1120 harboring pTP233 was transformed with the chloramphenicol (CM) resistance gene cassette flanked by the sequences homologous to the *yjeK* gene. Homologous recombination between chromosomal *yjeK* and the CM resistance cassette was confirmed by plating on LB agar containing 40 μg/mL CM, followed by diagnostic polymerase chain reaction (PCR). The CM resistance gene in the chromosome was removed by selecting a CM-susceptible strain after pCP20 plasmid introduction. Finally, the pCP20 plasmid was removed by culturing the CM-susceptible strain at 42°C, and deletion of *yjeK* and the CM cassette was further validated using diagnostic PCR. All primers used in strain construction and validation are listed in Table S2.

### Antibiotic Susceptibility Test

Bacterial antibiotics resistance was evaluated using the disk diffusion test on Mueller-Hinton agar (Becton Dickinson, Sparks, MD, USA) (21). Bacterial cells were grown in LB broth at 37°C for 16–18 h, and cells with an optical density at 600 nm (OD_600_) of 0.6–0.7, equivalent to 5 × 10^7^ colony forming units (CFU)/mL, were spread on Mueller-Hinton (MH) agar using sterile cotton swabs before positioning antibiotic disks (BBL™ Sensi-Disc™, Becton Dickinson). The following 10 different antibiotics were tested: amoxicillin/clavulanic acid (AMC) 30 μg, ampicillin (AM) 10 μg, cephalothin (CF) 30 μg, gentamicin (GM) 10 μg, kanamycin (K) 30 μg, nalidixic acid (NA) 30 μg, neomycin (N) 30 μg, ampicillin/sulbactam (SAM) 20 μg, sulfamethoxazole/trimethoprim (SXT) 25 μg, and tetracycline (TE) 30 μg. The diameter of the growth inhibition zone was used to determine antibiotic resistance.

### Motility Test

Bacterial swimming motility was assessed on semi solid-agar plates (0.3% agar) at 37°C as detailed previously (22). Each strain grown at OD_600_ of 0.6–0.7 was spotted onto the semi-solid agar and incubated at 37°C. The diameters of the swimming halos were measured at 4 and 12 h, respectively. All experiments were repeated three times.

### Analysis of Lipopolysaccharide (LPS) Profile

*Salmonella* strains were cultivated in LB broth medium for 16 h and LPS was extracted based on the hot phenol-water method (23). In brief, the bacterial cells were harvested by centrifuging at 10,000 × *g* for 20 min. The bacterial cell pellets were suspended in lysis solution (0.1 M sodium dodecyl sulfate (SDS), 50 mM Tris base, 0.128 M NaOH), incubated for 5 min, and treated with P/C/I solution (phenol:chloroform:isoamyl alcohol = 25:24:1). After heating at 65°C for 15 min, the separated aqueous phase was transferred to a new vial and mixed with 0.5 mL of 3 M Na-acetate (pH 5.6) and 17 mL of 95% ice-cold ethanol. LPS fractions were precipitated at −20°C overnight and centrifuged at 10,000 × *g* for 20 min. The precipitated LPS was resuspended in sterilized water and treated with DNase (Sigma-Aldrich) and RNase (Promega, Madison, WI, USA) at 37°C for 1 h. For LPS purification, the LPS solution was mixed with P/C/I solution and subjected to centrifugation at 10,000 × *g* for 20 min. The purified LPS was suspended in sterilized water and quantified using Pierce LAL chromogenic endotoxin quantitation kit (Thermo Scientific Inc., IL, USA). LPS samples of 5 μg were loaded on a 12% deoxycholate (DOC)-polyacrylamide gel electrophoresis (PAGE) gel (24) and the LPS profiles were visualized by silver staining (25).

### Analysis of Outer Membrane Proteins (OMPs)

Bacterial OMPs were isolated based on the method previously reported (26). Bacterial cells cultivated in LB broth were harvested by centrifugation at 2,500 × *g* for 20 min. The bacterial cell pellets were suspended in 10 mM N-2-hydroxyethylpiperazine N′-2-ethanesulfonic acid and subjected to sonication with Vibra-cell ultrasonic liquid processors (Sonics & Materials Inc., Newtown, CT, USA). The bacterial membrane fraction of cell lysates was collected by ultracentrifugation using a 45 Ti rotor (Beckman Coulter, Brea, CA, USA) at 100,000 × *g* for 1 h at 4°C. After ultracentrifugation, the membrane fraction was resuspended in a buffer containing 1% N-lauroylsarcosine solution (Sarkosyl, Sigma-Aldrich) to solubilize and remove inner membrane proteins. The sarkosyl-treated OMPs fraction was purified using ultracentrifugation as described above and finally resuspended in phosphate-buffered saline (PBS). The concentration of OMPs was measured using the Pierce BCA protein assay kit (Thermo Scientific Inc.). OMPs of 10 μg were analyzed using 12% SDS-PAGE and visualized by staining with Coomassie Brilliant Blue. The protein bands of interest were analyzed by the peptide mass fingerprinting method (27). In brief, the proteins on the SDS-PAGE gel were extracted and digested with trypsin (Promega). The digested peptides were mixed with α-cyano-4-hydroxycinnamic acid in 50% acetonitrile/0.1% trifluoroacetic acid (Sigma-Aldrich) and the peptide mixtures were analyzed using matrix-assisted laser desorption ionization-time-of-flight mass spectrometry (Microflex LRF 20, Bruker Daltonics, MA, USA) as previously described (27). Mass spectra were collected in the m/z range of 600–3,000 and internally calibrated by trypsin autodigestion peaks (m/z 842.510, 2211.1046). The mass spectra were analyzed using MASCOT server ver. 2.3 (Matrix Science, London, UK). Proteins with MASCOT scores >73 were considered to be significantly matched to the target proteins (*P* < 0.05).

### RNA Extraction and Quantitative Real-Time Reverse Transcription (qRT-PCR)

To prevent RNA degradation, bacterial cells cultivated in LB broth or animal cells infected with *Salmonella* were treated with RNAprotect Bacteria Reagent (Qiagen, Hilden, Germany) or RNAlater stabilization solution (Ambion, Austin, TX, USA) according to the manufacturer's instructions. Bacterial total RNAs were isolated using RNeasy mini kit (Qiagen) and residual chromosomal DNAs were removed using TURBO DNA-free kit (Ambion). RNA was reverse-transcribed into cDNA using RNA to cDNA EcoDry Premix with random hexamers (Clontech Laboratory, CA, USA), and cDNA corresponding to 10 ng of input RNA was used as a template in each qPCR. Primers were designed using Primer Express Software ver. 3.0 (Applied Biosystems, MA, USA) and their sequences are listed in Table S2. qPCR was conducted using a StepOnePlus real-time PCR instrument (Applied Biosystems) with SYBR green reagent (Power SYBR Green PCR Master Mix, Applied Biosystems) to detect the amplified PCR product. Relative expression levels of each gene were normalized to those of *gyrB* (28) and are presented as the average from three tests using independently extracted RNA samples.

### Invasion and Survival Assays

HeLa human epithelial cells and RAW264.7 murine macrophage cells were grown in Dulbecco's modified Eagle medium (DMEM) supplemented with 10% fetal bovine serum (FBS) and seeded in 24-well culture plates at a density of 1 × 10^5^ and 2 × 10^5^ cells/well, respectively. *Salmonella* cells cultivated in LB broth overnight were added to animal cells at a multiplicity of infection of 100. At 30 min after bacterial infection, the cells were washed three times with PBS and replenished with fresh DMEM containing gentamicin (100 μg/mL) for 1.5 h to remove extracellular bacteria. The medium was replaced with fresh DMEM containing 10 μg/mL gentamicin for the remainder of the infection period. For the invasion assay, the infected HeLa cells were lysed using 1% Triton X-100 at 2 h post-infection, and cell lysates were diluted and plated onto LB agar. In the survival assay, the infected RAW264.7 cells were lysed at 10 h post-infection and the intracellular bacteria were enumerated in the same manner described for the invasion assay.

### Animal Ethics

All animal experiments were performed according to the guideline on the international laws and policies (Guide for the Care and Use of Laboratory Animals, The National Academies Press, 8th edition) and were approved by the Institutional Animal Care and Use Committee of Kangwon National University (approval no. KW-160201-1).

### Mouse Infection Experiments

The half-maximal lethal dose (LD_50_) was calculated by the method of Reed and Muench (29). Six-week-old female BALB/c mice (Orient Bio Inc., Seongnam, Korea) were intraperitoneally (i.p.) injected with serially diluted bacterial cells at 10^2^ to 10^7^ CFU/mouse. The mice were monitored for 2 weeks after infection, and the numbers of dead mice were recorded to estimate LD_50_ values. Mice with sick and moribund signs were humanely euthanized. LD_50_ values were computed using the formula of log_10_ [50% end point] = A + (B × C), where A = log_10_ [infectious dose showing a mortality next below 50%], B = difference of logarithms = [50% – (mortality at infectious dose next below 50%)]/[(mortality next above 50%) – (mortality next below 50%)], and C = log_10_ [difference between serial infectious doses used in challenge studies. *In vivo* virulence tests were conducted using groups of four BALB/c mice that were i.p. infected with bacterial cells at 10^4^ CFU/dose. At 14 days post-infection (dpi), the mice were sacrificed, and the weights of the body and spleen were measured. The spleens were subsequently homogenized using TissueLyser II (Qiagen, USA) at 30 Hz for 1 min and the lysate was serially diluted and plated on *Salmonella-Shigella* (SS) agar (Difco, Becton Dickinson, MD, USA) for counting bacterial cells.

### Mouse Immunization and Challenge Experiments

The female BALB/c mice were divided into groups of six mice each and immunized with the wild-type and Δ*yjeK* strain by i.p. injection at 10^3^ CFU/dose and 10^4^ CFU/dose, respectively. PBS was used as a negative control for no immunization. After mouse immunization, blood was collected on days 0, 7, 14, 28, and 35, and the serum was stored at −20°C until use. After 28 days, the mice were orally challenged with ST2173 strain at 10^8^ CFU/dose (protection assay) or 10^10^ CFU/dose (survival assay). In the mouse protection assay, challenged mice were sacrificed 35 days after immunization, and the organs, including the liver and spleen, were collected and plated on SS agar. In the mouse survival assay, the challenged mice were monitored for an additional 24 days and the death rate was recorded.

### Enzyme-Linked Immunosorbent Assay (ELISA)

Specific IgG and IgM antibody responses in the serum were measured using ELISA as described previously (30). The optimal concentrations of serum and enzyme conjugates were determined by the checkerboard test (31, 32) using triplicate sera of immunized and non-immunized mice. The best binding ratios between serum and enzyme conjugates were selected to measure IgG, IgM, and IgG subclasses (IgG1 and IgG2a) antibody responses specific to *S*. Typhimurium. OMPs prepared from wild-type *S*. Typhimurium were added to microplates (Thermo Scientific Inc.) at 0.5 μg/well and incubated at 4°C overnight. The sera of immunized mice were diluted to 1:200 and added to the coated wells. After 1 h of incubation, the microplates were washed with PBS containing Tween 20 (PBST) and treated with horseradish peroxidase-labeled goat anti-mouse IgG (1:30,000 dilution; Bethyl Laboratories, TX, USA), anti-mouse IgM (1:35,000 dilution; Bethyl Laboratories), anti-mouse IgG1 (1:25,000 dilution, Bethyl Laboratories), or anti-mouse IgG2a (1:30,000 dilution, Bethyl Laboratories) secondary antibodies. Unbound secondary antibodies were washed with 0.05% PBST three times. The 3,3',5,5'-tetramethylbenzidine (Surmodics Inc., MN, USA) substrate was then added to each well and incubated for 5 min. The enzymatic reaction was stopped by the addition of 100 μL of H_2_SO_4_ at 0.5 M. Finally, the levels of antibodies were quantified by measuring color development at OD_450_ using an Epoch plate reader (BioTek, GA, USA).

For cytokine measurement, splenocytes were isolated from the spleens, counted, and plated at 2 × 10^5^ cells/well in complete medium, which consists of RPMI-1640 (GenDEPOT, TX, USA), 10 mM HEPES (Duchefa), and 10% FBS. The plated cells were treated with LPS (5 μg/mL, Sigma-Aldrich) or heat-killed *S*. Typhimurium cells (HKC; 10^8^ CFU/0.1 mL) and incubated at 37°C in 5% CO_2_ for 48 h. The supernatants of the cultured cells were collected and stored at −20°C until cytokine measurement. The concentrations of interferon (IFN)-γ, interleukin (IL)-6, and tumor necrosis factor (TNF)-α were determined using cytokine ELISA kits (MAX™ Standard, BioLegend, Inc., CA, USA) according to the manufacturer's instructions. The concentration of cytokines was calculated with Four Parameter Logistic Curve software (MyAssays Ltd., https://www.myassays.com/).

### Statistical Analysis

All values are expressed as means ± standard deviation. *P*-values were calculated using one- or two-way analysis of variance with Tukey's multiple comparison test. A *P* < 0.05 was considered statistically significant. All statistical tests were performed with GraphPad Prism 5 (GraphPad Software Inc., CA, USA).

## Results

### The Δ*yjeK* Mutant Shows Extensive Alternations in Bacterial Physiology

**Figure 1:**
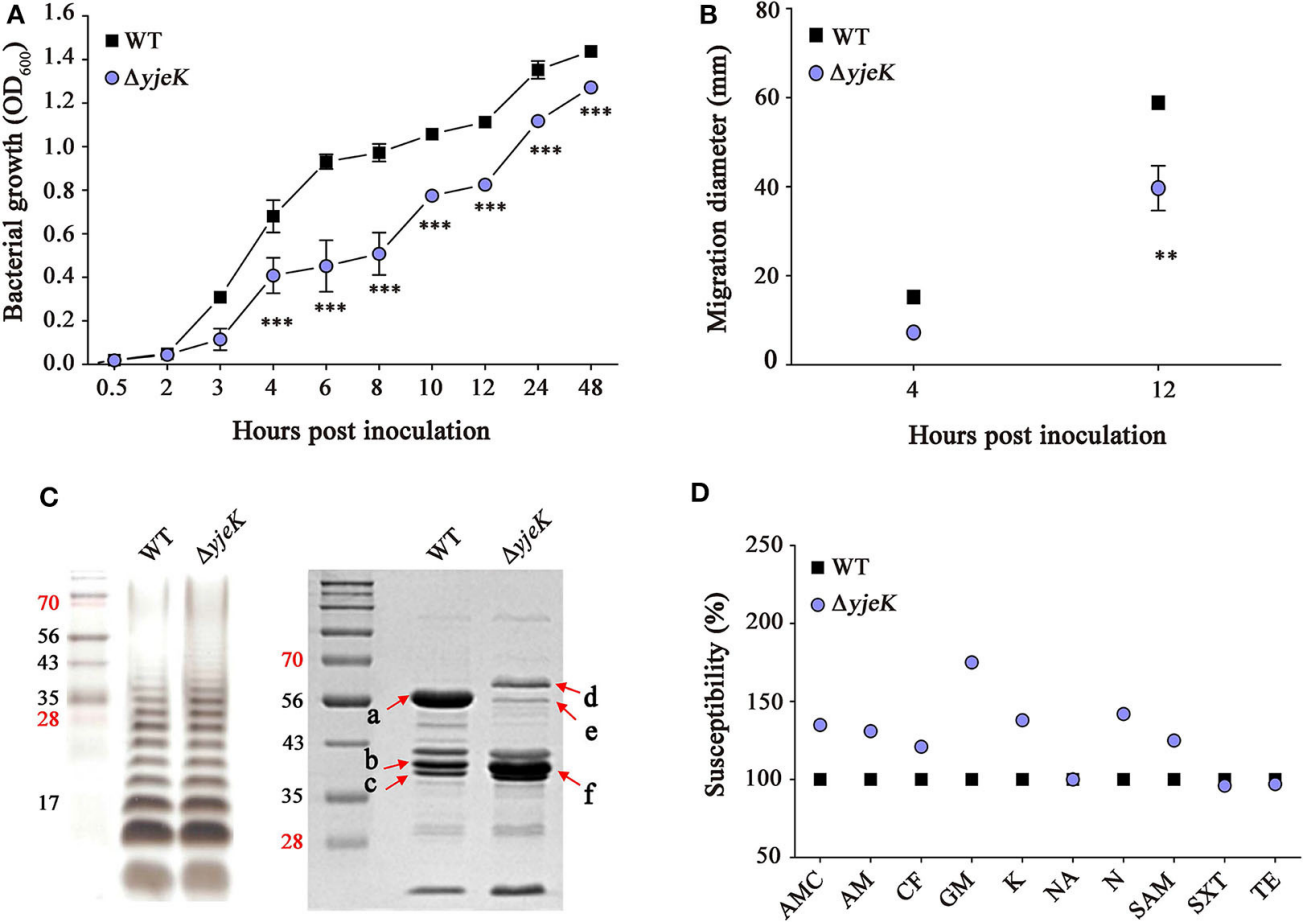
Characterization of the Δ*yjeK* mutant strain. **(A)** Growth curves of wild-type and the Δ*yjeK* mutant strains in LB broth (^**^P < 0.01; ^***^P < 0.001). **(B)** Swimming motility tests of wild-type and the Δ*yjeK* mutant strains. Diameters of bacterial swimming halos were measured on semi-solid agar plates at 4 and 12 h and analyzed by two-way analysis of variance (^**^P < 0.01). **(C)** Lipopolysaccharides (left) and outer membrane proteins (right) profiling in the wild-type and Δ*yjeK* mutant strains. Lane M indicates molecular weights (kDa). Protein bands indicated with arrows are identified in Table S4. **(D)** Antimicrobial susceptibility tests of the wild-type and Δ*yjeK* mutant strains in MH medium. Susceptibility of the wild-type strain to each antibiotic was set to 100%: amoxicillin/clavulanic acid (AMC), ampicillin (AM), cephalothin (CF), gentamicin (GM), kanamycin (K), nalidixic acid (NA), neomycin (N), ampicillin/sulbactam (SAM), sulfamethoxazole/trimethoprim (SXT), and tetracycline (TE). The tests were repeated twice in triplicate and the averaged values are plotted.

Two adjacent genes of *yjeK* (1,029 bp) and *efp* (567 bp) are transcribed in the opposite direction to each other with a distance of 40 bp between their start codons. A continuous sequence comprising the majority of *yjeK* (from 153 to 990 bp) was deleted in the chromosome of ST1120 using the λ red recombination method (19) (Figure S1A). Because the *efp* gene is located near the deletion site, the expression of *efp* was compared between the wild-type and deletion strains. Transcription of *efp* was hardly influenced by the deletion in the Δ*yjeK* mutant strain, ruling out the possibility of a polar effect on EF-P activity in the Δ*yjeK* strain (Figure S1B). The growth of the Δ*yjeK* strain was significantly delayed, and its motility was also dampened compared to those of the wild-type strain (Figures 1A,B). The analytical profile index (API) test showed that the Δ*yjeK* mutant strain shut down biochemical activities associated with arginine dihydrolase and lysine decarboxylase, indicating altered metabolic pathways in arginine and lysine decarboxylation in the mutant strain (Figure S2; Table S3).

LPS and OMP profiles showed that the Δ*yjeK* mutant strain exhibited increased abundance in the long O-antigen moieties and changed the levels of multiple proteins (Figure 1C). Six OMPs showing differential production between the wild-type and Δ*yjeK* mutant strains were identified by mass spectrometry (arrows in Figure 1C; Table S4). In accordance with the decreased motility of the Δ*yjeK* strain on agar plates, FliC flagellin production was remarkably decreased in the Δ*yjeK* mutant strain. In addition, two of the most abundant OMPs, OmpA, and OmpD, were predicted to show a change in expression in the Δ*yjeK* strain.

OmpA induces strong immunogenicity in *Salmonella* infection (33), and the porin OmpD is less produced to reduce bacterial permeability to oxidizing molecules and to facilitate bacterial survival in hosts (34, 35). Bacterial OMPs forming porins and efflux pumps influence bacterial resistance against antibiotics by decreasing permeability (porins) or by increasing drug export (efflux pumps). The observed increase in the porin OmpD in the Δ*yjeK* mutant therefore motivated us to further test its resistance against antibiotics. The Δ*yjeK* mutant strain was susceptible to all antibiotics tested except for NA, TE, and SXT (Figure 1D). Notably, the inhibition zones of GM, N, K, and AMC increased up to 77% (21.0 ± 0.0 mm), 45% (18.9 ± 0.5 mm), 36% (21.8 ± 1.1 mm), and 34% (27.5 ± 0.7 mm), respectively, in the mutant strain. Taken together, these results demonstrated that the Δ*yjeK* strain exhibited significant alterations in growth, motility, metabolism, and antibiotics resistance. These pleiotropic effects of YjeK on bacterial physiology have been also observed by Zou et al. (16) previously.

### The Δ*yjeK* Mutant Shows Attenuated Virulence *in vitro* and *in vivo*

**Figure 2:**
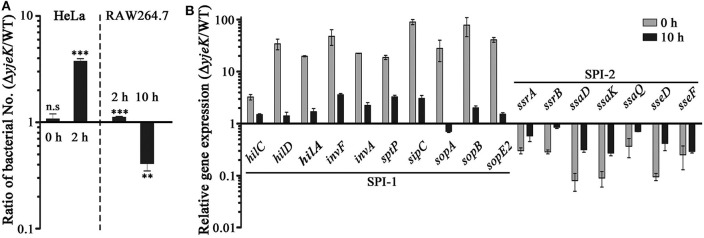
Virulence test of the Δ*yjeK* mutant strain in mammalian cells. **(A)** Epithelial HeLa cells (left) and macrophage RAW264.7 cells (right) were treated with the wild-type and Δ*yjeK* mutant strains at a multiplicity of infection of 100. Numbers of intracellular bacteria were counted at 2 and 10 h post-infection and the ratios between the two strains are plotted. Significant differences (^**^P < 0.01; ^***^P < 0.001) were determined by one-way analysis of variance (with Tukey's multiple comparison test); n.s. indicates not significant. **(B)** Expression of genes associated with SPI-1 and SPI-2 T3SSs was compared between the wild-type and Δ*yjeK* mutant strains in RAW264.7 cells using qRT-PCR. Total RNAs were extracted from bacterial cells used in the infection inoculums (0 h) and from infected RAW264.7 cells at 10 h post-infection. The mRNA levels of each test gene were normalized using those of the *gyrB* gene and the expression levels are presented as ratios between the two strains.

Navarre et al. previously reported that *S*. Typhimurium lacking *yjeK* or *yjeA* had attenuated virulence when infected to mice, and the absence of YjeA increased the expression of SPI-1 proteins using two-dimensional SDS-PAGE analysis (14). Accordantly, we also observed that the mRNA levels of SPI-1 genes were significantly increased in the Δ*yjeK* mutant strain, including genes encoding regulators, the secretion apparatus, and their effectors (Figure 2B). Effector proteins translocated by SPI-1 type III secretion system (T3SS) are important for *Salmonella* invasion into host cells, intracellular survival, and inflammatory response regulation (36). Besides, we also found that the Δ*yjeK* mutant decreased the expression levels of SPI-2 T3SS-associated genes (Figure 2B), which are critical for *Salmonella* survival and proliferation inside host cells (36). During the process of systemic infection, *Salmonella* fine-tunes the expression of multiple virulence effectors timely and spatially for its successful invasion into host cells and dissemination into other sites (36).

Given the differential expression of genes associated with SPI-1 and SPI-2 T3SSs between the wild-type and Δ*yjeK* mutant strains, we further investigated the ability of the bacteria to invade epithelial cells and survive inside macrophages. The Δ*yjeK* mutant entered host epithelial cells at a 3.73-fold greater rate than wild-type bacteria at 2 h post-infection (Figure 2A), but failed to fully proliferate inside macrophages at 10 h post-infection, showing a 0.41-fold decrease in intracellular numbers (Figure 2A). Inside macrophages, the mutant strain still showed altered expression in SPI-1/SPI-2 T3SSs-associated genes, with increased levels of SPI-1 and decreased levels of SPI-2 in comparison with those of the wild-type strain; however, this differential expression in the Δ*yjeK* mutant was alleviated during bacterial adaptation to the intracellular milieu (compare SPI-1 and SPI-2 expression in Figure 2B).

**Figure 3:**
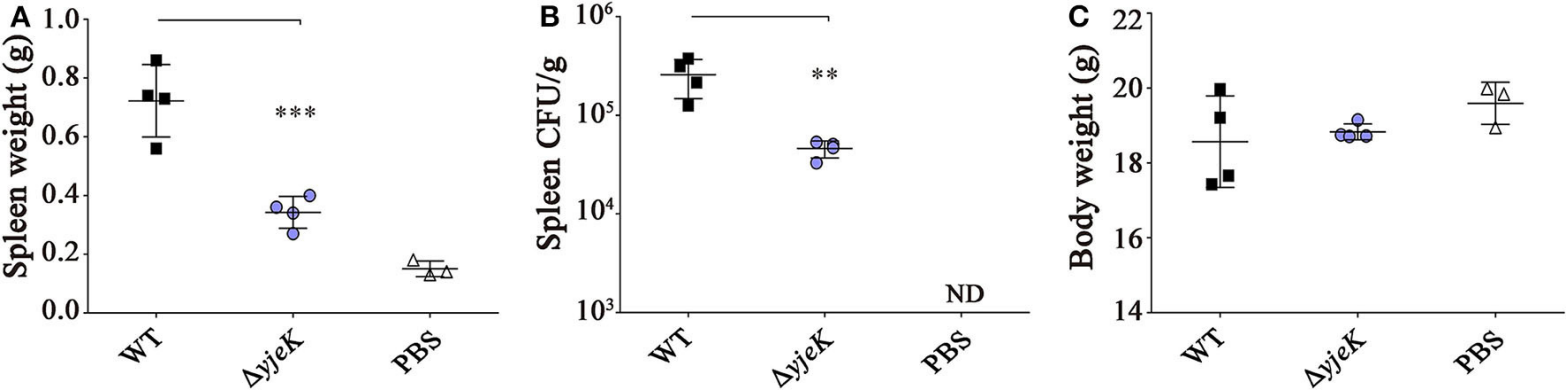
Virulence test of the Δ*yjeK* mutant strain in BALB/c mice. Groups of female BALB/c mice (*n* = 4) were i.p. injected with the Δ*yjeK* mutant strain (10^4^ CFU/mouse), wild-type strain (10^4^ CFU/mouse), or PBS (100 μL/mouse; control). **(A)** At 7 days post-infection, all mice were sacrificed, and the spleen was collected and weighed. **(B)** The spleen was homogenized and cultured on an SS agar plate to count bacterial numbers. **(C)** Body weights of infected mice were measured at euthanization and plotted. Significant differences (^**^P < 0.01; ^***^P < 0.001) were determined by one-way analysis of variance (with Tukey's multiple comparison test).

We further evaluated the virulence of the Δ*yjeK* mutant *in vivo* using mouse models of infection. The Δ*yjeK* mutant inoculated in BALB/c mice via the i.p. route showed ~1,000-fold attenuation in virulence with an LD_50_ value of 10^6.5^ CFU (compared with that of 10^3.2^ CFU of wild-type ST1120; Table 1). The clinical signs and bacterial burden of the mice infected with the Δ*yjeK* mutant were also clearly different from those of the wild-type infection group: the weight of the spleen was significantly lower after Δ*yjeK* mutant infection (Δ*yjeK*, 0.34 ± 0.05 g vs. wild-type, 0.72 ± 0.12 g), whereas *Salmonella*-induced splenomegaly was observed with wild-type strain infection (Figure 3A). Bacterial numbers in the spleen were also decreased in the Δ*yjeK* mutant infection group at 4.6 × 10^4^ CFU/g compared to 2.6 × 10^5^ CFU/g with infection of the wild-type strain (Figure 3B). However, the body weights of the mice in the two groups were comparable (Figure 3C). Overall, the lack of splenomegaly and reduced numbers of bacteria detected in the spleens of the mice infected with the Δ*yjeK* mutant strain confirmed its attenuated virulence *in vivo*.

**Table 1:** LD_50_ values of *S*. Typhimurium Δ*yjeK* mutant strain in BALB/c mice.

**Strains**	**Infection dose (CFU/mouse)**	**Number of dead mice/ total mice number**	**LD_**50**_**
Wild-type	10^5^	3/4	10^3.2^
	10^4^	3/4	
	10^3^	0/4	
	10^2^	0/4	
Δ*yjeK*	10^7^	3/4	10^6.5^
	10^6^	0/4	
	10^5^	0/4	
	10^4^	0/4	

### Immunization With the Δ*yjeK* Mutant Stimulates Immune Responses in Mice

**Figure 4:**
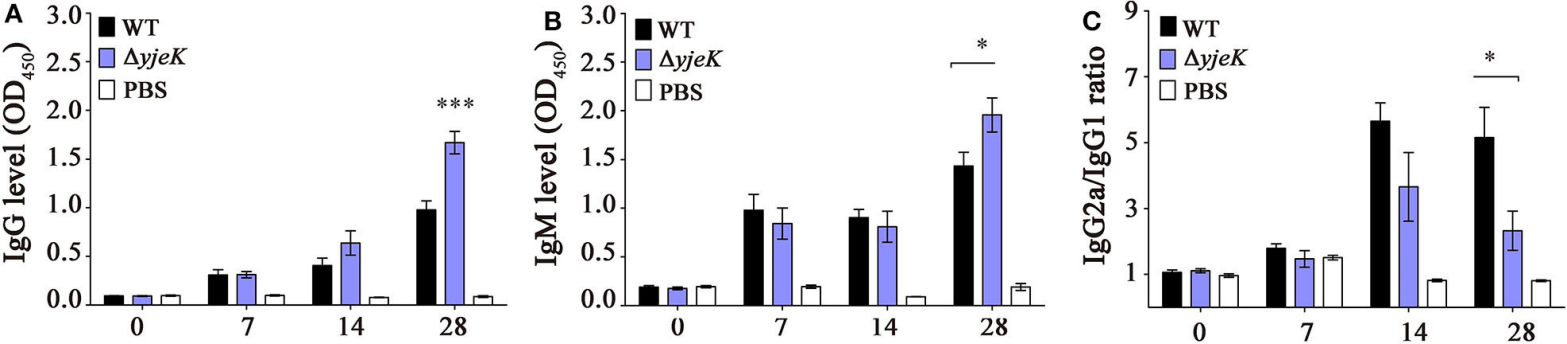
Production of *Salmonella*-specific antibodies in mouse sera. Levels of **(A)** IgG, **(B)** IgM, and **(C)** IgG subclasses (IgG2a and IgG1) were measured in the serum of BALB/c mice (three groups of *n* = 6 each) immunized i.p. with the Δ*yjeK* mutant strain (10^4^ CFU/mouse), wild-type strain (10^3^ CFU/mouse), or PBS (100 μL/mouse; negative control). Mice sera were collected at 0, 7, 14, and 28 days post-immunization and serum antibodies were quantified by ELISA. The levels of IgG subclasses are presented in ratios between IgGa2 and IgG1. Data of antibody levels are shown as mean ± SEM; significant differences (^*^P < 0.05; ^***^P < 0.001) were determined by one-way analysis of variance (with Tukey's multiple comparison test).

The immunologic effect of the Δ*yjeK* mutant was evaluated by measuring the levels of serum antibodies produced after infection in BALB/c mice. The levels of IgG and IgM antibodies were comparable between the groups infected with the wild-type and Δ*yjeK* mutant strains until 14 dpi, but notably increased by immunization with the Δ*yjeK* mutant strain at 28 dpi by 1.7- and 1.5-fold, respectively, compared to those induced by wild-type *Salmonella* at 28 dpi (Figures 4A,B). The IgG2a/IgG1 ratio of immunized mice was measured, which is commonly used as a surrogate marker for immune balance between T helper (Th)1 and Th2 responses against microbial infection (37, 38). The IgG2a/IgG1 ratios were in the range of 2.32 (28 dpi) to 3.66 (14 dpi) in the mice immunized with the Δ*yjeK* mutant, and the ratios from immunization with wild-type *Salmonella* were between 5.16 (28 dpi) and 5.65 (14 dpi) (Figure 4C). This result indicated that both the wild-type and mutant strains stimulated the Th1-mediated immune response more vigorously than the Th2-mediated immune response in BALB/c mice. Considering that the Δ*yjeK* mutant induced higher levels of IgG but lower IgG2a/IgG1 ratios than the wild-type strain, we inferred that immunization with the Δ*yjeK* mutant conferred not only IgG2a-associated protection but also IgG1-associated protection more intensively than possible with wild-type *Salmonella*.

**Figure 5:**
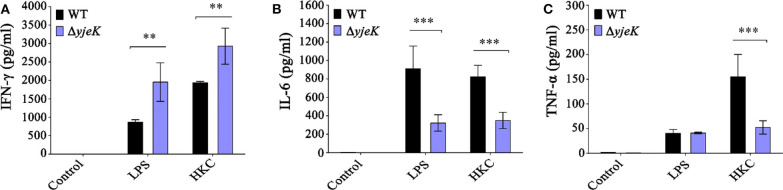
Stimulation of cytokines production in mice immunized with the Δ*yjeK* mutant. Groups of female BALB/c mice (*n* = 4) were i.p. injected with the Δ*yjeK* mutant strain (10^4^ CFU/mouse) or wild-type strain (10^3^ CFU/mouse). At 28 days post-immunization, the mice were euthanized and splenocytes were collected. The splenocytes were stimulated with lipopolysaccharide (LPS), heat-killed *S*. Typhimurium cells (HKC), or medium as a control. After 48-h stimulation, the concentrations of **(A)** IFN-γ, **(B)** IL-6, and **(C)** TNF-α secreted from the splenocytes were measured using capture ELISA. Cytokine assays were conducted in triplicate and data of cytokines production are shown as the mean concentration ± SEM; significant differences (^**^P < 0.01; ^***^P < 0.001) were determined by one-way analysis of variance.

After immunization, the spleens of the mice were harvested at 28 dpi, and splenocytes isolated from the spleens were treated with *S*. Typhimurium LPS and HKCs to estimate the levels of cytokine production against *Salmonella* immunogens. Three different cytokines, IFN-γ, IL-6, and TNF-α, were all increased in response to LPS or HKCs, regardless of the bacterial strains used in immunization (Figure 5). Interestingly, immunization with the Δ*yjeK* mutant promoted IFN-γ production against LPS (2.24-fold) and HKCs (1.5-fold) more significantly than that observed with wild-type *Salmonella* (Figure 5A). However, the production of pro-inflammatory cytokines IL-6 and TNF-α was induced to a lower degree after immunization with the Δ*yjeK* mutant strain for both the LPS and HKCs treatments (Figures 5B,C). Considering that murine splenocytes consist of a variety of cell populations, including B cells, T cells, natural killer (NK) cells, and others, the distribution of tested cytokines might be influenced by cell types other than T cells. In this context, Ramarathinam et al. showed that NK cells were the primary lymphoid cells responsible for IFN-γ production in the spleen of *Salmonella*-infected mice (39). Overall, these results indicated that mice immunized with the Δ*yjeK* mutant induced IgG and IgM antibodies proficiently without systemic infection signs in host organs, and concomitantly modulated the production of cytokines against challenges with immunogenic antigens of *Salmonella*.

### Immunization With the Δ*yjeK* Mutant Protects Mice Against *Salmonella* Infection

**Figure 6:**
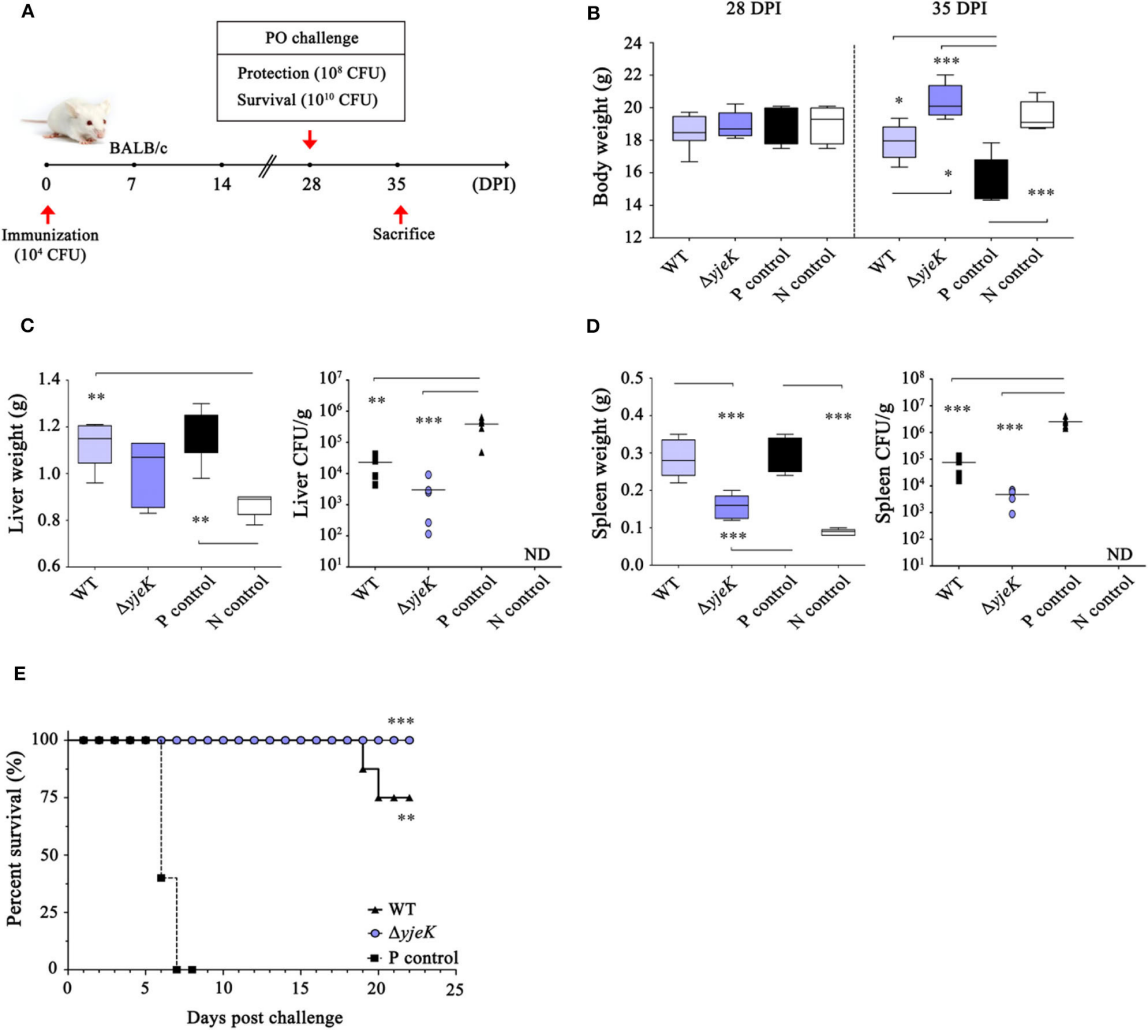
Prophylactic effect of immunization with the Δ*yjeK* mutant in mice. **(A)** Groups of six BALB/c mice each were i.p. injected with the Δ*yjeK* mutant (10^4^ CFU/mouse), wild-type strain (10^3^ CFU/mouse), or PBS (100 μL/mouse; N control) as a negative control. At 28 days post-immunization (dpi), the mice were orally challenged with a wild-type *S*. Typhimurium strain (ST2173; AM^R^) at a dose of LD_50_ (10^8^ CFU/mouse). Positive (P) control mice were administered PBS and then challenged with ST2173 at the same dose. At 7 days post-challenge (35 dpi), the mice were sacrificed and the weight of the **(B)** body, **(C)** liver, and **(D)** spleen was measured. The mouse liver and spleen were homogenized and cultured on SS agar plates containing AM (150 μg/mL). **(C,D)** The bacterial burden (CFU/g) in the organs was determined and normalized using the liver/spleen weight. Significant differences (^*^P < 0.05; ^**^P < 0.01; ^***^P < 0.001) were determined by one-way analysis of variance (with Tukey's multiple comparison test). **(E)** Survival of immunized mice against lethal *Salmonella* infection. Adult female BALB/c mice (*n* = 8 per group) were i.p. injected with the Δ*yjeK* mutant (10^4^ CFU/mouse) and orally challenged with ST2173 at a dose of 100LD_50_ (10^10^ CFU/mouse) at 28 dpi. the survival of the mice was monitored daily. The *P*-value was determined by a log-rank (Mantel-Cox) test (^***^P < 0.001).

The prophylactic effect by immunization with the Δ*yjeK* mutant was evaluated in BALB/c mice. After i.p. immunization with the candidate vaccine Δ*yjeK* mutant strain, the mice were orally infected with wild-type *S*. Typhimurium 2173 (ST2173) at 28 dpi (Figure 6A). The typical signs of *Salmonella* infection, including a rough hair coat, crusted and closed eyes, loss of appetite, hunched posture, and shivering, weight loss, and death, were observed in mice infected with ST2173 without pre-immunization, and the body weight was also reduced to around 77% by ST2173 infection at 35 dpi (see the positive challenge control in Figure 6B). However, mice immunized with the Δ*yjeK* mutant instead showed an increase in body weight for 7 days after ST2173 infection, showing similar body weights to those of healthy mice not injected with any *Salmonella* strain (Figure 6B). Moreover, there was no sign of an enlarged liver and spleen in mice immunized with the candidate vaccine, whereas the challenged mice without pre-immunization had enlarged livers and spleens with 1.35- and 3.4-fold increased weights, respectively, compared to those of healthy mice (Figures 6C,D). The bacterial burden of the liver and spleen was also significantly mitigated in the mice immunized with the candidate vaccine (3.0 × 10^3^ CFU/g liver and 4.8 × 10^3^ CFU/g spleen) in comparison with those of mice immunized with wild-type *Salmonella* (2.3 × 10^4^ CFU/g liver and 7.5 × 10^4^ CFU/g spleen) and the non-immunized mice (positive challenge control: 3.9 × 10^5^ CFU/g liver and 2.5 × 10^6^ CFU/g spleen) (Figures 6C,D).

The prophylactic efficacy of pre-infection with the candidate vaccine was further evaluated by monitoring the survival rates of immunized mice after oral infection of ST2173 with a bacterial dose of 100 LD_50_ (Figure 6E). Most of the challenge control mice that were not immunized with any *Salmonella* strains succumbed to death within 1 week after ST2173 infection. However, all of the mice immunized with the candidate vaccine survived the ST2173 infection during the monitoring period. Mice pre-infected with wild-type *Salmonella* showed 75% survival against challenge with virulent strain at 24 dpi. Overall, these results indicated that the candidate vaccine could offer complete protection against *Salmonella* infection.

## Discussion

Non-typhoidal serovars of *Salmonella enterica* generally cause self-limiting gastroenteritis in infected humans, but some serovars may penetrate the intestinal epithelia and become disseminated to systemic sites such as the liver and spleen by entering the lymphatic and blood circulatory systems. This type of invasive *Salmonella* infection is commonly ascribed to serovars such as *S*. Typhimurium, *S*. Enteritidis, and *S*. Dublin (40, 41). Infection by iNTS results in clinical signs and symptoms of fever, hepatosplenomegaly, and/or respiratory complications, which may be fatal in immunocompromised individuals. The increased risk of iNTS diseases in recent decades has promoted vaccine development targeting the most common iNTS-associated serovars. In view of the immune responses to administered vaccines, live vaccines with long-lasting and strong antibody responses are highly recommended (42). However, a live vaccine strain needs to be sufficiently attenuated in virulence without losing its immunogenicity and should not revert to virulent derivatives. A variety of *Salmonella* mutant strains have been suggested as candidate vaccines, including auxotrophic mutants defective in aromatic compounds and purine biosynthesis (43), mutants lacking global transcription regulators (44), and mutants defective in signal molecules synthesis (45). The Δ*yjeK* mutant strain with extensive physiological pleiotropy could be considered as another candidate vaccine against iNTS diseases.

In *Salmonella enterica*, YjeK was first demonstrated to influence *Salmonella* virulence and resistance to antimicrobial compounds, operating in a common pathway coordinated by YjeK, YjeA, and EF-P (14). The YjeK and YjeA proteins, or their homologs, are linked to each other and cooperate to activate EF-P by β-lysylation (11, 15). EF-P is a fourth elongation factor in addition to three universal elongation factors, EF-Tu, EF-Ts, and EF-G, and is required for diverse translation processes, including formation of the first peptide bond of N-formylmethionyl-initiated peptides (12, 13, 46), formation of polyphenylalanine peptides programmed with poly(rU) (47), and prevention of ribosome stalling on consecutive proline codons (48). Therefore, it is reasonable to expect extensive changes in bacterial phenotypes in the absence of YjeK and/or YjeA.

In this study, we demonstrated that YjeK could influence the production and expression of multiple virulence determinants implicated in successful host infection. LPS and OMPs which are localized on the outer surface of bacterial envelope and play a role as representative immunogenic antigens showed alterations in their compositional abundance in the Δ*yjeK* mutant strain. Structural modifications at the O-antigen moiety of LPS enable bacteria to evade the host immune response and also render bacteria more susceptible or more resistant to antimicrobial agents. Bacterial OMPs forming porins and efflux pumps can also affect bacterial resistance against antimicrobial agents produced in host. Besides, the expression of virulence effectors was changed depending on the presence of YcfR. *Salmonella* translocates more than 30 virulence effectors into the host cytosol with the aid of two specialized secretion systems called SPI-1 and SPI-2 T3SSs. Effectors delivered by SPI-1 T3SS promote cytoskeletal rearrangements for *Salmonella* invasion into host cells, and induce the proinflammatory responses and maturation of *Salmonella*-containing vacuole (SCV) inside host cells. By contrast, the effectors secreted by SPI-2 T3SS predominantly play roles inside host cells, including SCV maturation and trafficking, downregulation of inflammatory responses, and apoptosis of infected cells (36, 49). Effectors production and translocation are controlled temporally and spatially in response to the extracellular environments encountered during *Salmonella* infection. Therefore, a *Salmonella* strain defective in fine-tuning the activities of SPI-1 and SPI-2 T3SSs will fail to manipulate the host immune system, resulting in its dampened virulence inside hosts. Indeed, we showed that the Δ*yjeK* mutant strain had significantly increased expression levels of genes encoding SPI-1 T3SS components and its cognate secretion effectors (Figure 2). Increased production and subsequent translocation of SPI-1 T3SS effectors might facilitate the invasion of the Δ*yjeK* mutant strain in contact with host enterocytes, which might in turn stimulate the release of cytokines and host inflammatory responses, leading to antibody production and T-cell activation. In addition, the decreased expression of genes associated with SPI-2 T3SS in the Δ*yjeK* mutant strain (Figure 2B) might prolong bacterial persistence inside phagocytes without aggressive apoptosis, and eventually delaying rapid bacterial clearance in hosts.

Naïve CD4+ T cells are activated into two subsets of Th1 and Th2 cells in response to antigen stimulation. Th1 cells secrete IL-2 and INF-γ, which promote cell-mediated immune responses, while cytokines secreted by Th2 cells trigger humoral immune responses, including antibody production (37, 50). Mice immunized with the Δ*yjeK* mutant strain produced more IgG and IgM antibodies in the serum than those immunized with wild-type *Salmonella* but showed a lower IgG2a/IgG1 ratio, which is indicative of the type of immune responses elicited between Th1 and Th2-mediated responses (51). The antibody abundance and IgG subclass distribution by the Δ*yjeK* mutant strain demonstrate that Th2 cells-mediated immune responses could develop to confer successful humoral immunity after immunization with the Δ*yjeK* mutant strain in mice. Finally, 100% of the mice immunized with the Δ*yjeK* mutant strain were protected from lethal doses of *Salmonella* infection, without signs of *Salmonella* systemic infection (Figure 6).

Collectively, this study highlights the possibility of the Δ*yjeK* mutant strain as an ideal live attenuated vaccine against iNTS infection.

## Data Availability Statement

All datasets generated for this study are included in the article/Supplementary Material.

## Ethics Statement

The animal study was reviewed and approved by the Institutional Animal Care and Use Committee of Kangwon National University (approval no. KW-160201-1).

## Author Contributions

SP, BJ, HY, and T-WH conceived, designed, and coordinated the study. SP, BJ, and EK performed the experiments and interpreted the data. SP, S-TH, HY, and T-WH wrote the manuscript. All authors took part in discussing the results and in reviewing the manuscript.

## Conflict of Interest

The authors declare that the research was conducted in the absence of any commercial or financial relationships that could be construed as a potential conflict of interest.
