# Supplementary material for: Salmonella Typhimurium Lacking YjeK as a Candidate Live Attenuated Vaccine Against Invasive Salmonella Infection
Source: Front Immunol. 2020 Jun 23;11:1277. doi: 10.3389/fimmu.2020.01277 (PMC7324483; doi:10.3389/fimmu.2020.01277)
Supplement: Supplementary file 1 [file Data_Sheet_1.docx]

Supplementary Material

*Salmonella* Typhimurium lacking YjeK as a candidate live attenuated vaccine against invasive *Salmonella* infection

Park et al.

**Supplementary Table S1. Bacterial strains and plasmids used in this study**

| **Strains** | **Relevant characteristics** | **References** |
| --- | --- | --- |
| ST1120 | Wild-type *S.* Typhimurium isolated in Korea | Kim et al., 2017 |
| ST2160 | ST1120 derivative (Δ*yjeK*::FRT, Cm^R^) | This study |
| ST2161 | ST2160 derivative (Δ*yjeK*) | This study |
| ST14028 | ATCC14028 | ATCC |
| ST2173 | ST14028 with plasmid pBBR1-MCS4 (AM^R^) | This study |
| **Plasmids** | **Characteristics** |  |
| pTP233 | λ-Red recombinase, temperature sensitive replication (TE^R^) | Poteete and Fenton, 1984 |
| pKD3 | FRT-Cm-FRT cassette (Cb^R^ Cm^R^) | Datsenko and Wanner, 2000 |
| pCP20 | FLP recombinase, temperature sensitive replication (AM^R^) | Cherepanov and Wackernagel, 1995 |
| pBBR1-MCS4 | Broad-host-range cloning vector (AM^R^) | Truong, et al. 2014 |

**Supplementary Table S2. Primers used in this study**

| **Primers** | **Sequence (5’-3’)** |
| --- | --- |
| *yjeK*_long F | TTAGCACTGCCGAAGCTGTAAATCCAGCGGTGTTTTGCTTGTGTAGGCTGGAGCTGCTTCGAAG |
| *yjeK*_long R | GATGAAAACCTCCGGGCAGGACAGGACGCCAGGCGCTTGTTCATATGAATATCCTCCTTAGTTCCT |
| *yjeK*_com F | ATTAGCACTGCCGAAGCTGTA |
| *yjeK*_com R | CGAGAAGATTGGTTAGCGCAAC |
| *hilA* qPCR F | GCTGCACCAGGAAAGCATTAAG |
| *hilA* qPCR R | CGAAGTCCGGGAATACATCTGA |
| *hilC* qPCR F | GCCGCTGAAGAGGTGAGTTTTA |
| *hilC* qPCR R | AATATTTCCAGCCCCCATACG |
| *hilD* qPCR F | GCTGTTCCTGCTTACTGCTTTTC |
| *hilD* qPCR R | AATGTTGTAAACGCGCTCCTTT |
| *invA* qPCR F | ACAAAACATATGCTGGACCAACTG |
| *invA* qPCR R | ACGCTGCAAAACTTCAGATATACG |
| *invF* qPCR F | GCGGAAAAGCGAAGAGTGAA |
| *invF* qPCR R | AACGGCTAATTGGGTGATGTTC |
| *sipC* qPCR F | CGAAGGGATGAATGCGTTGT |
| *sipC* qPCR R | GCAGCCCCTTATATTCCAGTTTG |
| *sopA* qPCR F | TACGTCACAAAGCCAACCTCTCT |
| *sopA* qPCR R | GTGGCATTTGCAGCCAGATA |
| *sopB* qPCR F | GCAATGATTTACGCCCTGAAG |
| *sopB* qPCR R | TGGCGCGCTAATGTATTGAC |
| *sopE2* qPCR F | CGGAGAAGGACATTTTTGCAA |
| *sopE2* qPCR R | TGGGTAGTCGGTATGTCTGTTTGT |
| *sptP* qPCR F | GGCGATACAAAAGTGGCAGAA |
| *sptP* qPCR R | CGCAGGCTATTTCCATCCAT |
| *ssaD* qPCR F | GGTGGAATGGGTGTCCTGTTAA |
| *ssaD* qPCR R | CGCCCGCACATAATGAATATT |
| *ssaK* qPCR F | GAGGCATTGATGCGAGAAACT |
| *ssaK* qPCR R | CGGCATATCGTGTTGAGGAA |
| *ssaQ* qPCR F | CATCCTGCAGTAATCTACCACATCA |
| *ssaQ* qPCR R | AAACCCGTTGGCCATGTAAA |
| *sseD* qPCR F | ATGCGCAGCTATAACGTAGAAAAA |
| *sseD* qPCR R | TGCTCTAAACGCTTCATCAATTG |
| *sseF* qPCR F | GGTTGCTGCAGCGGTAATTT |
| *sseF* qPCR R | CACAGCAAGCATCCCCAATA |
| *ssrA* qPCR F | GGTTCAGAAACGGCAGCATATAA |
| *ssrA* qPCR R | GTTCAGCTTCTTCAAACCGTTGA |
| *ssrB* qPCR F | TCTGTTAGCGGCATTGCAAA |
| *ssrB* qPCR R | GGTCGTGTCAGCGTTTAATTCA |
| *yjeK* qPCR F | ACTGGTCGCCCGCTTTG |
| *yjeK* qPCR R | CGCCTCGTCCACTTCATTAG |
| *efp* qPCR F | CGATTTTCGTTCCGGTCTTAA |
| *efp* qPCR R | CCTGGCCTTTACCCGGTTT |
| *yjeA* qPCR F | AAAACGTGCGGCGATTATG |
| *yjeA* qPCR R | ACTCATGCAGGGCGTCTCA |
| *gyrB* qPCR F | TCGCTCAGCAGTTCGTTCAT |
| *gyrB* qPCR R | GATTGCGGTGGTTTCCGTAA |

**Supplementary Table S3. Analysis of biochemical phenotypes of *S.* Typhimurium Δ*yjeK* mutant strain**

| **Biochemical phenotypes** | **Strain characteristics** | |
| --- | --- | --- |
|  | **Wild-type** | **Δ*yjeK*** |
| Acetoin production | − | − |
| Amygdalin fermentation | − | − |
| Arabinose fermentation | + | + |
| Arginine dihydrolase^*^ | + | − |
| Citrate utilization | + | + |
| β-Galactosidase | − | − |
| Gelatinase | − | − |
| Glucose fermentation | + | + |
| H2S production | + | + |
| Indole production | − | − |
| Inositol fermentation | + | + |
| Lysine decarboxylase | + | **−** |
| Mannitol fermentation | + | + |
| Melibiose fermentation | + | + |
| Ornithine decarboxylase | + | + |
| Rhamnose fermentation | + | + |
| Sorbitol fermentation | + | + |
| Sucrose fermentation | − | − |
| Tryptophan deaminase | − | − |
| Urease | − | − |

* Differential phenotypes of the Δ*yjeK* mutant strain compared to WT are underlined.

**Supplementary Table S4.** **Identification of OMPs with different expressions between wild-type and Δ*yjeK* mutant strains**

| **Spots** | **Identified proteins** | **Gene symbols** | **Protein sequence coverage [%]** |
| --- | --- | --- | --- |
| a | flagellin FliC | *fliC* | 73 |
| b | porin OmpD | *ompD* | 80 |
| c | OmpA | *ompA* | 47 |
| d | flagellin FljB | *fljB* | 47 |
| e | flagellin FliC | *fliC* | 42 |
| f | porin OmpD | *ompD* | 69 |


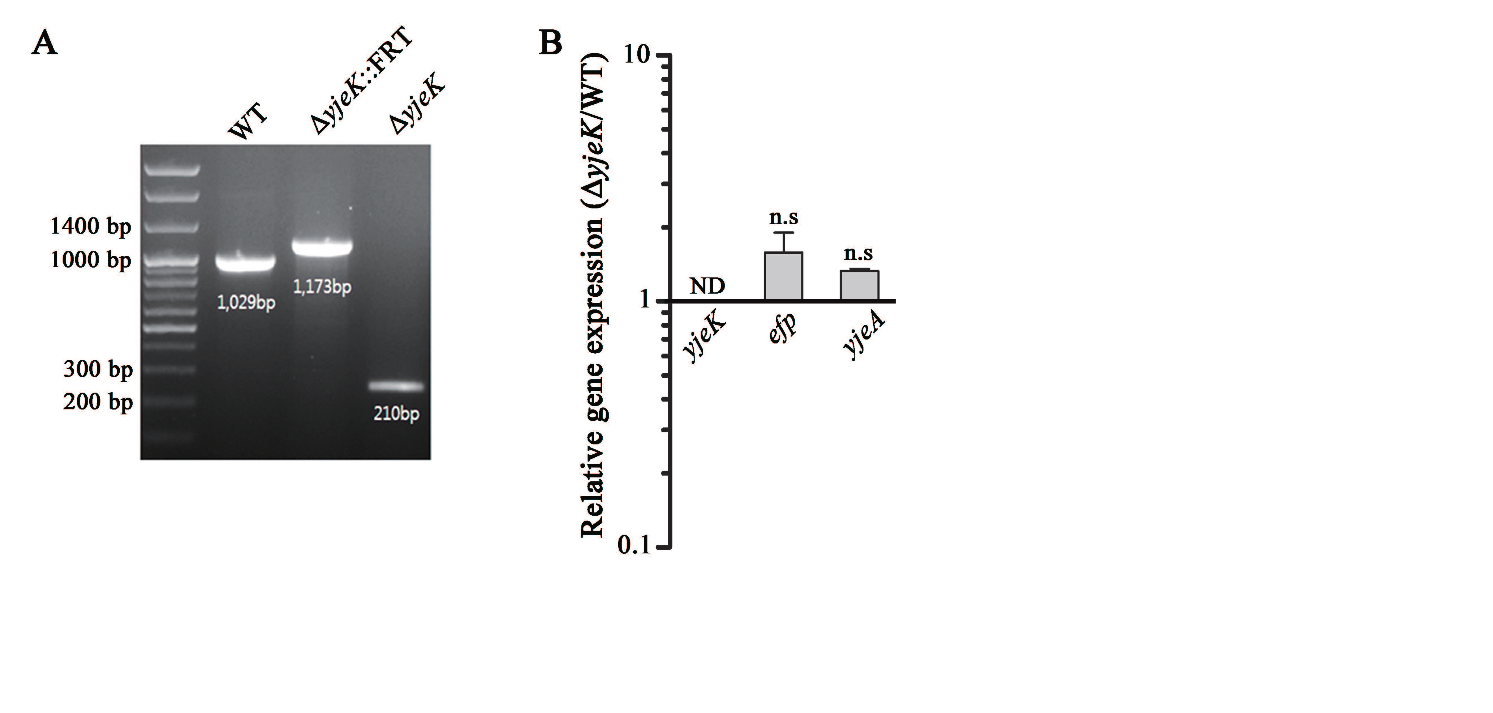


**Supplementary Figure S1.** Construction of a Δ*yjeK* mutant strain. (A) Deletion of *yjeK* (1029 bp) sequences from 153 bp to 990 bp was validated by diagnostic PCR using primers binding to sequences adjacent to the *yjeK* gene. (B) The mRNA levels of *yjeK*, *efp*, and *yjeA* were compared between the wild-type and Δ*yjeK* mutant strains using qRT-PCR. Transcription levels of each gene were normalized using those of the *gyrB* gene. N/D indicates not detected; n.s. means not significant.

**
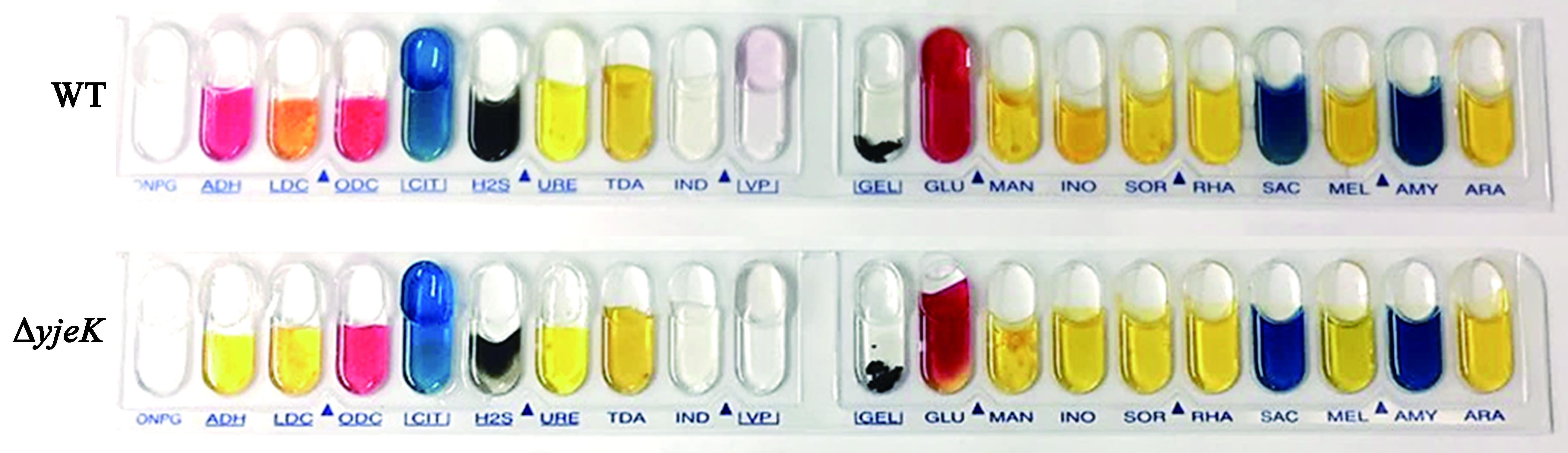
**

**Supplementary Figure S2.** Analytical profile index (API) test of the Δ*yjeK* mutant strain.

Bacterial cells in suspension medium were added to 20 separate compartments of the API 20E biochemical test strips and the reaction results were interpreted after 20-h incubation.

**References**

Kim, S., Kim, E., Park, S., Hahn, T.-W., and Yoon, H. (2017). Genomic Approaches for Understanding the Characteristics of Salmonella enterica subsp. enterica Serovar Typhimurium ST1120, Isolated from Swine Feces in Korea. J. Microbiol. Biotechnol 27**,** 1983-1993.

Poteete, A. R., & Fenton, A. C. (1984). λ red-dependent growth and recombination of phage P22. Virology 134, 161-167.

Datsenko, K.A., and Wanner, B.L. (2000). One-step inactivation of chromosomal genes in Escherichia coli K-12 using PCR products. Proc Natl Acad Sci U S A 97**,** 6640-6645.

Cherepanov, P. P., & Wackernagel, W. (1995). Gene disruption in Escherichia coli: TcR and KmR cassettes with the option of Flp-catalyzed excision of the antibiotic-resistance determinant. Gene 158, 9-14.

Truong, Q.L., Cho, Y., Park, S., Park, B.-K., and Hahn, T.-W. (2016). Brucella abortus mutants lacking ATP-binding cassette transporter proteins are highly attenuated in virulence and confer protective immunity against virulent B. abortus challenge in BALB/c mice. Microb. Pathog. 95**,** 175-185. doi: 10.1016/j.micpath.2016.04.009.
